# O.6.2-5 Data infrastructure Sport and Physical Activity: the creation of a thematic classification for indicators

**DOI:** 10.1093/eurpub/ckad133.275

**Published:** 2023-09-11

**Authors:** Marjolein Duijvestijn, Wanda Wendel-Vos

**Affiliations:** National Institute for Public health and the Environment, The Netherlands; marjolein.duijvestijn@rivm.com; National Institute for Public health and the Environment, The Netherlands; marjolein.duijvestijn@rivm.com

## Abstract

Accurate facts and figures concerning physical activity are crucial to inform policy decisions. In the Netherlands, a vast amount of data related to this domain is currently available. However, accessing the required information is increasingly challenging. Therefore, the National Institute for Public Health and the Environment (RIVM), in collaboration with several key knowledge institutes, initiated a project to integrate existing data on sport and physical activity into a thematic data-infrastructure.

The key knowledge institutes involved in this project are NOC*NSF, Mulier Institute, the Knowledge Center for Sport and Physical Activity, RIVM, the Dutch Knowledge Center for Injury Prevention, and Statistics Netherlands. These organizations have been responsible for collecting data on 20 key-indicators for sports and physical activity since 2015. Having experts in the domain of sports and physical activity (e.g. research, practice, policy) endorse the thematic data structure was crucial. In total, 99 experts from 27 organizations across the domains of research, practices, and policy were involved in the project. An extensive inventory of existing and new indicators was carried out by the team in collaboration with the experts. The process involved multiple online and live sessions, as well as two consultations with online forms for each theme, resulting in the identification of 12 themes and nearly 400 indicators. These indicators were categorized within each theme based on the relevance assigned by the experts.

In addition, the position of the current set of 20 Key-Indicators was assessed within each theme. Results showed that the set of Key-Indicators was indeed largely still up-to-date. Nevertheless, there is reason to more closely examine and evaluate the current set in the near future.

In the future RIVM will continue to coordinate the sports and physical activity data-infrastructure in the Netherlands by advocating and facilitating harmonized data collection at the national and local level.

